# Comparative analysis of Doxycycline and Ayurvedic herbs to target metastatic breast cancer: An *in-silico* approach

**DOI:** 10.37796/2211-8039.1448

**Published:** 2024-06-01

**Authors:** Acharya Balkrishna, Rashmi Mittal, Rohan Malik, Hariom Verma, Kuldeep Singh Mehra, Hariom Chaturvedi, Okeshwar, Swami Ishdev, Ajay Kumar, Vedpriya Arya

**Affiliations:** a Patanjali Herbal Research Department, Patanjali Research Institute, Haridwar, India; b Department of Yog Science, University of Patanjali, Haridwar, India; c Department of Sanskrit, University of Patanjali, Haridwar, India

**Keywords:** MMP9, Phytochemicals, Doxycycline, Molecualr docking, MD-Simulation

## Abstract

**Background:**

Metastasis of breast cancer cells to distant sites including lungs, liver, lymph node, brain and many more have substantially affected the overall survival outcome and distant metastasis free survival rate amongst the diseased individuals. Several pre-clinical and clinical studies were carried out to determine the potency of vigorous inhibitors but they extensively deteriorated the patient’s quality of life. Hence, there exists an urgent need to explore potent natural remedy to fight against metastatic breast cancer.

**Methods:**

Ayurvedic medicinal plants documented in literature for their ability to fight against breast cancer was screened and their respective active moieties were evaluated to exert inhibitory effect against MMP9. Drug like efficacy of phytochemicals were determined using Molecular docking, MD Simulation, ADMET and MM-PBSA and were further compared with synthetic analogs i.e. Doxycycline.

**Results:**

Out of 1000 phytochemicals, 12 exerted highest binding affinity (BA) even more than −9.0 kcal/mol that was significantly higher in comparison to Doxycycline which exhibited BA of −7.3 kcal/mol. In comparison to 37 × 30 × 37 Å, 53 × 45 × 66 Å offered best binding site and the highest BA was exhibited by Viscosalactone at LYS104, ASP185, MET338, LEU39, ASN38. During MD Simulation, Viscosalactone-MMP9 complex remained stable for 20 ns and the kinetic, electrostatic and potential energies were observed to be better than Doxycycline. Furthermore, Viscosalactone obtained from *Withania somnifera* justified the Lipinski’s Rule of 5.

**Conclusion:**

Viscosalactone obtained from *W. somnifera* may act as promising drug candidate to fight against metastatic breast cancer.

Metastatic breast cancer (MBC), commonly referred to as “secondary breast cancer” (SBC), describes the spread of breast cancer cells to other areas of the body through the lymphatic or blood systems [1]. The disease can spread to visceral organs like the liver, lung, and brain, causing widespread and potentially fatal metastases, or it can remain localized and to some extent it can metastasize to the bone as well. Since MBC is incurable, the main objectives of treatment are to maintain quality of life while prolonging life and managing symptoms. A family of proteases known as matrix metalloproteinases (MMPs) is highly upregulated in breast cancer (BC) and has a variety of biological roles in the initiation and spread of cancer [2]. Gelatinase B, or MMP9, is a protein cleaver and important player in the remodelling of the extracellular matrix (ECM). It is also linked to tumor invasion, metastasis, and the alteration of the tumor microenvironment. Type IV collagen is one of the collagens that MMP9 can break down. This type of collagen contributes to the breakdown of the basement membrane, which facilitates migration, invasion, and metastases [3]. MMP9 is secreted as a pro-enzyme that is not active, and the crucial step in controlling it is activating latent MMP9. The increased expression of MMP9 in human and experimental cancer models, both *in vitro* and *in vivo*, is associated with the progression of the tumor [4–6]. In Breast cancer, MMP9 is linked to poor prognostic factors and short-term survival outcomes at both the proteomic and transcriptomic levels. MMP9 may be crucial for the advancement of BC in both stromal and tumor cells [7].

Ayurveda, an ancient Indian medical system that dates back to the ancient ages is a method of identifying disease and applying a variety of therapies and methods to combat it [8]. Complex cancer cases can be significantly treated with Ayurvedic medicine without experiencing negative side effects. The Ayurvedic practitioner performs specific examinations to diagnose cancer, following which appropriate treatment is recommended. Cancer patients’ bodies can be cleansed of toxins and free radicals with the aid of Ayurvedic treatment [9,10]. It alters the environment of cancer cells and strengthens and restores the function of various organs. Ayurvedic medications boost immunity, which further kills cancer cells.

Several phytochemicals found in Ayurvedic herbs demonstrated a range of anti-cancer activity, primarily in the formof anti-oxidant, anti-inflammatory, anti-mutagenic, and apoptosis inducing properties that may stop cancer from developing in its early stages [11,12]. The aim of the present study is to evaluate the potency of Ayurvedic herbs to target metastasis in breast cancer through *in-silico* studies. Computational drug analysis has proved to be highly efficacious in screening of potential drug candidates against both the acute and chronic disorders. Docetaxel, a dietary phytochemical is currently under Phase-II clinical trial videNCT00852332 asmentioned on clinicaltrials.gov against HER2-breast cancer. Before proceeding with the clinical trial, the phytochemical was evaluated using *in-silico* approaches as evident from the study conducted by Rajagopal *et al.* in 2020 and several others, and based on its inhibitory potential the results were further validated using *in vitro* and *in-vivo* studies [13]. Henceforth, it can be depicted that *in-silico* studies plays a vital role in initial screening of drug candidates to fight against BC and several others. In present study, the efficacy of Ayurvedic herbs was also compared with the synthetic analog named as Doxycycline. Although Doxycycline is well-known for exerting antitumor activities and the study conducted by Huijun *et al.* in 2023 also unleashed its potency to retard breast cancer bone metastasis [14]. Wherein the administration of Doxycycline significantly affected the circulating levels of MMPs and TIMP2 in bone micro-environment and therefore inhibited the metastasis of BC cells to bones. Despite of its anti-breast cancer activities, side effects induced by the drug cannot be ignored. Henceforth, there exists an urgent need to look forward for natural therapeutic regimes to fight against the disease.

## 1. Materials and methods

### 1.1. Preparation of phytochemicals library and receptors

The phytochemicals of 100 Ayurvedic plants that have been shown to be effective in the literature in the fight against breast cancer were obtained from the PubChem and ChEMBL databases. From these 100 plants, a total of 1000 phytochemicals were screened. 3D structure of phytochemicals was fetched using Chimera in .pdb format. Compounds without a .mol/.sdf were drawn and transformed using the Structure File Generator and Online SMILES Translator. Crystal structure of Matrix Metalloproteinase 9 was retrieved from www.rcsb.org with PDB ID of 1L6J [12,15].

### 1.2. Molecular docking study

On a 64-bit platform, the AutoDock Vina PyRx software was used for molecular docking. The prepared phytochemical file was uploaded once the prepared protein had been loaded with the specified parameters. The original ligand, which refers to all atoms within a 5 Å radius, was set as the binding site. The phytochemicals were converted from .mol2/.mol/.sdf to .pdbqt format, while the target receptor was converted from .pdb to .pdbqt format. PyRx software, which is used for virtual screening and docking in AutoDock Vina, was then used for multidrug docking [16–19].

### 1.3. ADMET analysis

Using the ADMETlab Web Server, the drug library was divided into clusters for Absorption BioMedicine Distribution Metabolism Excretion (ADME) analysis in order to identify the best drug compounds that were soluble, druggable, lead-like, and nonviolating. For every phytochemical, an input file containing the SMILES (Simplified Molecular-Input LineEntry System) was obtained from PubChem and ChEMBL [20].

### 1.4. Determination of active site involved in interaction

The position of the binding between the ligand and receptor was ascertained using the UCSF Chimera. Through docked structure analysis of ligands with MMP9, researchers were able to obtain a better understanding of the significance of particular amino acids in ligand-receptor interaction. Using the command line, amino acid residues involved in interactions within 5 Å were shown. Additional analyses were conducted using the commercially available synthetic analog, Doxycycline. Each naturally occurring moiety and synthetic analogue’s targeted MMP9 active site was studied.

### 1.5. Molecular dynamic simulation and calculation of MM-PBSA

The best-docked complexes were subjected to 20 ns of molecular dynamics simulations using NAMD-VMD software installed on Windows 11. To compute forces and energies, the CHARMM 22 parameter force-field was utilized. A fixed temperature of 310 K and a simulated time of 20 ns were employed for the best run. In order to detect any potential modifications in the protein-ligand dynamics complexes, the simulated complexes were further assessed and visualized using VMD after the experiment was completed. To ascertain the stability of simulated complexes, electrostatic, potential, and kinetic energy have been computed and projected onto their trajectories using the VMD and NAMD software.

## 2. Results & discussion

### 2.1. Preparation of 3D structure of ligands and receptors

The canonical SMILES that were obtained from PubChem were used to obtain the 3D structure of ligands via the RBPS Web Portal. As a result, the corresponding 3D structures could be retrieved and converted into monomeric units in the .pdf and .mol2 formats. All the 3D structure of phytochemicals and the synthetic inhibitor Doxycycline was saved in .pdb format. The 3D structure of MMP9 with PDB ID 1L6J, which was downloaded from the RCSB Web Portal and saved in .pdb format, was analyzed using UCSF Chimera. The AutoDock Tool was utilized to conduct molecular docking at multiple receptor sites in order to ascertain the binding affinities and active site. In PDBQT form, the energy of both the ligands and receptors was reduced and preserved.

### 2.2. Molecular docking study

The AutoDock tool was used to dock ligands with MMP9. The molecules that were docked with the highest binding pocket occupancy and the lowest Gibbs free energy were ranked based on their bond angle. The binding affinities (BA) of the ligands docked with MMP9 at the two distinct grid sites 53 × 45 × 66 Å and 37 × 30 × 37 Å are displayed in Kcal/mol. Docked postures with negligible binding affinity (less than 5 kcal/mol) were not considered for further investigation. Only ten phytochemicals out of a thousand were found to exhibit discernible inhibitory activity. In comparison to both the grid points, at 53 × 45 × 66 Å highest BA was observed. Acacetin, Apigenin, Chrysin, Cyanidin, Galangin, Kaempferol, Luteolin, Physagulin, Quercetin, Viscosalactone, Withanolide, and Withoxyloactone were found to present highest binding with MMP9 of −9.1, −9.6, −9.5, −9.7, −9.2, −9.1, −9.5, −9.7, −9.7, −9.8, −9.0, −9.5 kcal/mol respectively whereas Doxycycline, a synthetic inhibitor of MMP9 showcased BA of −7.3 kcal/mol. Highest BA was observed by Viscosalactone of −9.8 kcal/mol at 53 × 45 × 66 Å of MMP9. If any efficient drug candidate wants to target MMP9 in order to combat breast cancer, they can view the active site as an effective target (Table 1) [21].

### 2.3. Molecular dynamics simulation and calculation of free energy

Using NAMD software, an MD simulation of MMP9 was performed. As a synthetic inhibitor, Doxycycline was employed. The MD simulation was performed at 310 K for 20 ns. MMP9 complex with Acacetin, Apigenin, Chrysin, Cynadine, Galangin, Kaempferol, Luteolin, Physagulin, Quercetin, Viscosalactone, Withanolide and Withoxylactone respectively was evaluated for the stability of complex using the MD simulation for 20 ns. The outcomes demonstrated that for 20 ns, MMP-Viscosalactone complex remained to be highly stable even in comparison to the above mentioned other phytochemicals. Trajectory analysis makes clear that Viscosalactone showed behaviours resembling those of the synthetic analog Doxycycline and remained stable throughout the simulation process. Calculations of kinetic, potential, and electrostatic energy were also carried out to evaluate the molecules’ binding affinities. It was found that the results of the molecular docking study and the simulation study were concordant. Compared to their synthetic counterparts, every naturally occurring metabolite used in the study produced better outcomes (Table 2).

### 2.4. Determination of MMP9 active site involved in interaction

**Fig. 1:**
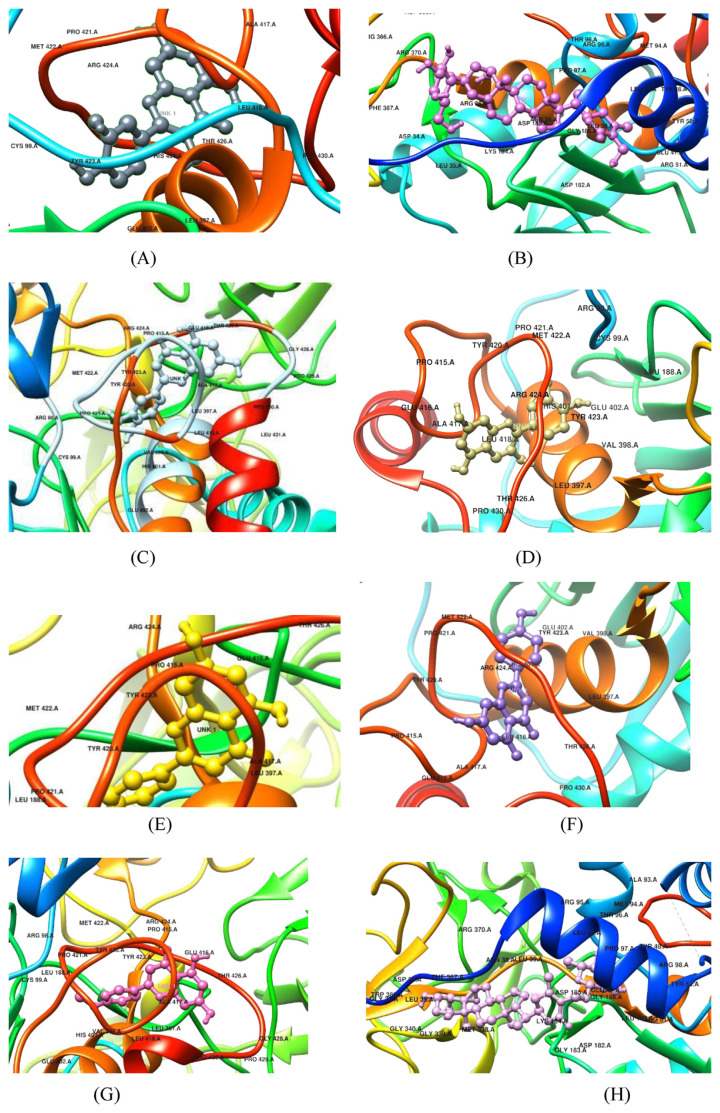
Figure representing interaction of phytochemicals and doxycycline with MMP9 at various active sites; (A): MMP9-Quercetin; (B) MMP9- Physagulin; (C) MMP9-Luteolin; (D) MMP9-Cynadine; (E) MMP9-Chrysin; (F) MMP9-Apigenin; (G) MMP9-Acaetin; (H) MMP9-Viscosalactone.

With the aid of UCSF Chimera, the likely MMP9 ligand binding site was identified. To determine which of the many amino acids was responsible for the interaction with the receptor, a region within 5 Å of where each ligand had chosen to bind to the receptor domain and the amino acid residues composing that area have been anticipated. MMP9 at 53 × 45 × 66 Å grid points created the binding site using Acacetin, Apigenin, Chrysin, Cyanidin, Galangin, Luteolin, Physagulin, Quercetin, Viscosalactone, and others comprising TYR423, ALA417, VAL398, LEU397, GLU 416; LEU418, ARG424, HIS401, TYR423, GLU402; TYR423, LEU397, ALA417, TYR420, GLU416; ARG424, HIS401, LEU418, TYR423, GLU402; TYR423, TYR420, PRO415, PRO421, LEU397; ASP185, LEU39, GLY186, ARG370, ASN38; THR426, LEU418, TYR423, HIS401, PRO421; LYS104, ASP185, MET338, LEU39, ASN38 respectively. Whereas with doxycycline it forms the binding site at ARG36, ASP34, ASR368, GLY367, PHE31. All the above mentioned phytochemicals exhibited better binding affinity with MMP9 in comparison to doxycycline. From these results it is evident that these phytochemicals can target MMP9 in an effective manner in comparison to doxycycline (Fig. 1).

### 2.5. ADMET

Based on the ADMET (Absorption, Dissolution, Metabolism, Excretion, Toxicity) model, the biological and physiochemical properties of the selected probable MMP9 inhibitors was screened using ADMETlab Web Server. Viscosalactone was further examined for its drug like biological activities after demonstrating the greatest inhibitory potential. Log Papp Caco2 absorption value, human intestinal absorption, plasma protein binding distribution value, blood protein barrier, CYP IA2 and CYP 3A4 inhibitor metabolism value, hepatotoxicity value, and mutagenicity values were determined. The ADMETlab drug capability test was passed by both the synthetic analog and the natural moiety (Table 3) [22].

## 3. Conclusion

Ayurvedic medicinal plants exhibited significant potency to fight against acute and chronic diseases. Their active constituents may emerge as a profound mono-therapeutic or combinational therapeutic approach to fight against MBC. The results of the present study indicated that active constituents of *Withania somnifera* may emerge as vital drug candidate to inhibit MMP9 to fight against MBC. The findings of the present study need to be further validated by *in vitro* and *in vivo* studies to introduce a potential drug to target MMP9.

## Figures and Tables

**Table 1:** Table representing binding affinities of phytochemicals and synthetic analogs at 53 × 45 × 66 Å & 37 × 30 × 37 Å with MMP9.

Interaction	Binding Affinity (Kcal/mol) (53 × 45 × 66 Å)	Binding Affinity (Kcal/mol) (37 × 30 × 37 Å)
MMP9-Acacetin	−9.1	−6.5
MMP9-Apigenin	−9.6	−6.8
MMP9-Chrysin	−9.5	−6.5
MMP9-Cynadine	−9.7	−6.8
MMP9-Galangin	−9.2	−6.5
MMP9-Kaempferol	−9.1	−6.9
MMP9-Luteolin	−9.5	−6.9
MMP9-Physagulin	−9.7	−7.4
MMP9-Quercetin	−9.7	−6.8
MMP9-Viscosalactone	−9.8	−8.4
MMP9-Withanolide	−9.0	−8.5
MMP9-Withaoxylactone	−9.5	−7.6
MMP9-Doxycycline	−7.3	−6.4

**Table 2:** Table representing binding energies computed by MM-PBSA during MD Simulation.

Target	Ligand	Energy Components (Kcal/mol)		

		Electrostatic	Potential	Kinetic
MMP9	Viscosalactone	−116107	−100992	1972.43
	Doxycycline	−12187.13	−4896.33	−4470.68

**Table 3:** ADMET prediction of phytochemicals and doxycycline.

Compound	Absorption		Dissolution	Metabolism		Excretion	Toxicity	
				
	Caco-2 Permeability	Pgp-inhibitor	PPB	CYP2C19 inhibitor	CYP1A2 inhibitor	T1/2	Skin Sensitization	Carcinogenicity
Acacetin	−4.834	—	97.23%	+++		0.696	++	–
Apigenin	−4.847	—	97.26%	+	+++	0.856	+++	–
Chrysin	−4.874	—	98.03%	++	+++	0.787	+++	-
Cyanidin	−5.344	—	95.43%	—	++	0.928	+++	—
Luteolin	−5.028	—	95.44%	–	+++	0.898	+++	—
Physagulin	−5.241	—	87.53%	—	—	0.088	—	–
Quercetin	−5.204	—	95.50%	—	+++	0.929	+++	—
Somniferine	−5.305	+++	72.93%	—	—	0.054	++	++
Viscosalactone	−5.415	–	52.32%	—	—	0.112	—	—
Withoxylactone	−5.095	–	43.23%	—	—	0.148	—	-
Doxycycline	−4.694	++	88.42%	+++	+++	0.884	+++	+

## Abbreviations

MMP9: Matrix Metalloproteinase-9
MBC: Metastatic Breast Cancer
ECM: Extracellular Matrix
HER2: Human Epidermal Growth Factor Receptor 2
PDB: Protein Data Bank
LGA: Lamarckian Genetic Algorithm
MD Simulation: Molecular Dynamic Simulation
VMD: Visual Molecular Dynamic
RMSD: Root Mean Square Deviation
ADMET: Absorption, Dissolution, Metabolism, Excretion, Toxicity.

## References

[1] Park M Kim D Ko S Kim A Mo K Yoon H Breast cancer metastasis: mechanisms and therapeutic implications Int J Mol Sci 2022 23 6806 10.3390/ijms23126806. 35743249 PMC9224686

[2] Mittal R Chaudhry N Pathania S Mukherjee TK Mechanistic insight of drug resistance with special focus on iron in estrogen receptor positive breast cancer Curr Pharmaceut Biotechnol 2014 15 1141 57 10.2174/1389201015666141126121240. 25429654

[3] Saikia M Retnakumari AP Anwar S Anto NP Mittal R Shah S Pillai KS Balachandran VS Peter V Thomas R Anto RJ Heteronemin, a marine natural product, sensitizes acute myeloid leukemia cells towards cytarabine chemotherapy by regulating farnesylation of Ras Oncotarget 2018 9 18115 27 10.18632/oncotarget.24771. 29719594 PMC5915061

[4] Riggio AI Varley KE Welm AL The lingering mysteries of metastatic recurrence in breast cancer Br J Cancer 2021 124 13 26 10.1038/s41416-020-01161-4. 33239679 PMC7782773

[5] Joseph C Alsaleem M Orah N Narasimha PL Miligy IM Kurozumi S Elevated MMP9 expression in breast cancer is a predictor of shorter patient survival Breast Cancer Res Treat 2020 182 267 82 10.1007/s10549-020-05670-x. 32445177 PMC7297818

[6] Balkrishna A Mittal R Arya V Potential role of miRNA in metastatic cascade of triple-negative breast cancer Curr Cancer Drug Targets 2021 21 153 62 10.2174/1568009620999201103201626. 33155912

[7] Muraleedharan A Kumar S Mittal R Pre-clinical and clinical evidence of recent therapeutic trends and spotting possibility of cure in near future Balkrishna A Therapeutic drug targets and phytomedicine for triple negative breast cancer Netherlands Bentham Science 2023 73 98 10.2174/9789815079784123010007

[8] Küpeli Akkol E Bardakci H Barak TH Aschner M Şeker Karatoprak G Khan H Herbal ingredients in the prevention of breast cancer: comprehensive review of potential molecular targets and role of natural products Oxid Med Cell Longev 2022 2022 6044640 10.1155/2022/6044640. 36017236 PMC9398845

[9] Balkrishna A Mittal R Arya V Stumbling blocks in reinvigorating the health of diseased individuals through herbal medicine Balkrishna A Therapeutic drug targets and phytomedicine for triple negative breast cancer Netherlands Bentham Science 2023 198 207 10.2174/9789815079784123010013.

[10] English DP Roque DM Santin AD HER2 expression beyond breast Cancer: therapeutic implications for gynecologic malignancies Mol Diagn Ther 2013 17 85 99 10.1007/s40291-013-0024-9. 23529353 PMC3660991

[11] Dash MK Joshi N Gautam DN Jayakumar R Tripathi YB Ayurvedic supportive therapy in the management of breast cancer J Herb Med 2021 29 100490 10.1016/j.hermed.2021.100490.

[12] Pettersen EF Goddard TD Huang CC Couch GS Greenblatt DM Meng EC UCSF Chimera—a visualization system for exploratory research and analysis J Comput Chem 2004 25 1605 12 10.1002/jcc.20084. 15264254

[13] Ponnulakshmi R Rekha UV Padmini R Perumal S Saravanan R Vishnupriya V Molecular docking analysis of docetaxel analogues as duel lipocalin 2 inhibitors Bioinformation 2020 16 438 43 10.6026/97320630016438. 32884206 PMC7452746

[14] Zhao H Pond G Simos D Wang Z Robertson S Singh G Doxycycline-induced changes in circulating MMP or TIMP2 levels are not associated with skeletal-related event-free or overall survival in patients with bone metastases from breast cancer Cancers 2023 15 571 10.3390/cancers15030571. 36765529 PMC9913061

[15] Mittal R Chaudhry N Mukherjee TK Targeting breast cancer cell signaling molecules PI3K and Akt by phytochemicals Cannabidiol, Nimbin and Acetogenin: an in silico approach J Biomed 2018 3 60 3 10.7150/jbm.25815.

[16] Balkrishna A Mittal R Sharma G Arya V Computational insights of phytochemicaldriven disruption of RNA-dependent RNA polymerase-mediated replication of coronavirus: a strategic treatment plan against coronavirus disease 2019 New Microbes New Infect 2021 41 100878 10.1016/j.nmni.2021.100878. 33815808 PMC8010343

[17] Gallivanone F Cava C Corsi F Bertoli G Castiglioni I In silico approach for the definition of radiomiRNomic signatures for breast cancer differential diagnosis Int J Mol Sci 2019 20 5825 10.3390/ijms20235825. 31756987 PMC6929037

[18] Lamichhane S Rai RP Khatri A Adhikari R Shrestha BG Shrestha SK Screening of phytochemicals as potential anti-breast cancer agents targeting HER2: an in-silico approach J Biomol Struct Dyn 2023 41 897 911 10.1080/07391102.2021.2014972. 34957911

[19] Ferreira LG Dos Santos RN Oliva G Andricopulo AD Molecular docking and structure-based drug design strategies Molecules 2015 20 13384 421 10.3390/molecules200713384. 26205061 PMC6332083

[20] Benet LZ Hosey CM Ursu O Oprea TI BDDCS, the Rule of 5 and drugability Adv Drug Deliv Rev 2016 101 89 98 10.1016/j.addr.2016.05.007. 27182629 PMC4910824

[21] Morris GM Huey R Lindstrom W Sanner MF Belew RK Goodsell DS AutoDock4 and AutoDockTools4: automated docking with selective receptor flexibility J Comput Chem 2009 30 2785 91 10.1002/jcc.21256. 19399780 PMC2760638

[22] Ali M Wani SU Salahuddin M Sn M Mruthunjaya K Dey T Recent advance of herbal medicines in cancer-a molecular approach Heliyon 2023 9 13684 10.1016/j.heliyon.2023.e13684. PMC9971193 36865478

